# Rupture of the Left Common Iliac Artery During Percutaneous Transluminal Angioplasty

**DOI:** 10.7759/cureus.106270

**Published:** 2026-04-01

**Authors:** Grant N Garrison, Ashwin Jagadish, Girendra Hoskere

**Affiliations:** 1 Internal Medicine, East Tennessee State University James H. Quillen College of Medicine, Johnson City, USA; 2 Critical Care, Ballad Health Medical Associates Pulmonology and Sleep, Bristol, USA

**Keywords:** aortoiliac rupture, chronic limb-threatening ischemia, common iliac artery, device malfunction, percutaneous transluminal angioplasty

## Abstract

This report aims to discuss a case of aortoiliac rupture during percutaneous transluminal angioplasty (PTA). The incidence of this type of rupture during PTA is low, specifically in the external iliac artery (EIA). We present the case of a 57-year-old male, a former smoker, with a history of metastatic cancer, who underwent PTA for severe limb-threatening ischemia. He experienced rupture in the left common iliac artery (CIA), which was promptly ligated. Emphasis is initially placed on examining the interplay between the lesion and the device, factors leading to the complication. Further discussion explores preprocedural imaging and alternative endovascular procedures, including primary stents, surgical grafts, and lithotripsy.

## Introduction

Percutaneous transluminal angioplasty (PTA) with selective stenting uses a balloon for dilation, and a stent is placed if necessary. In contrast, primary stenting involves placement of a stent without prior balloon dilation [1]. In this case, the patient experienced rupture during PTA with selective stenting. Complications such as occlusion, hematoma, and intimal damage have a low incidence in PTA [2]. According to *Vascular Specialist International*, the incidence of rupture was estimated to be 2.5%, all occurring in the external iliac artery (EIA) [3]. The smaller nature of the artery contributes to its vulnerability to rupture. Data are limited for ruptures of the larger common iliac artery (CIA), despite heavier calcification. The complexity of the lesion and the device used in PTA are the key factors considered to have led to the occurrence of CIA rupture.

## Case presentation

This is the case of a 57-year-old male, a former smoker, with a history of metastatic squamous cell lung cancer who underwent cardiologist-performed PTA for severe chronic limb-threatening ischemia (CLTI). The patient went to the ED in April of 2025 with right foot pain. According to the patient, the wounds developed over the course of a week. No medical evaluation had been done prior to this presentation to verify the duration of the wounds. There were two amputations done on the right foot. The first was the amputation of the third, fourth, and fifth toes for gangrene on May 5, 2025. Recovery was complicated by osteomyelitis of the fifth metatarsal and persistent gangrene. He had a fifth transmetatarsal amputation with debridement on July 8, 2025.

**Figure 1:**
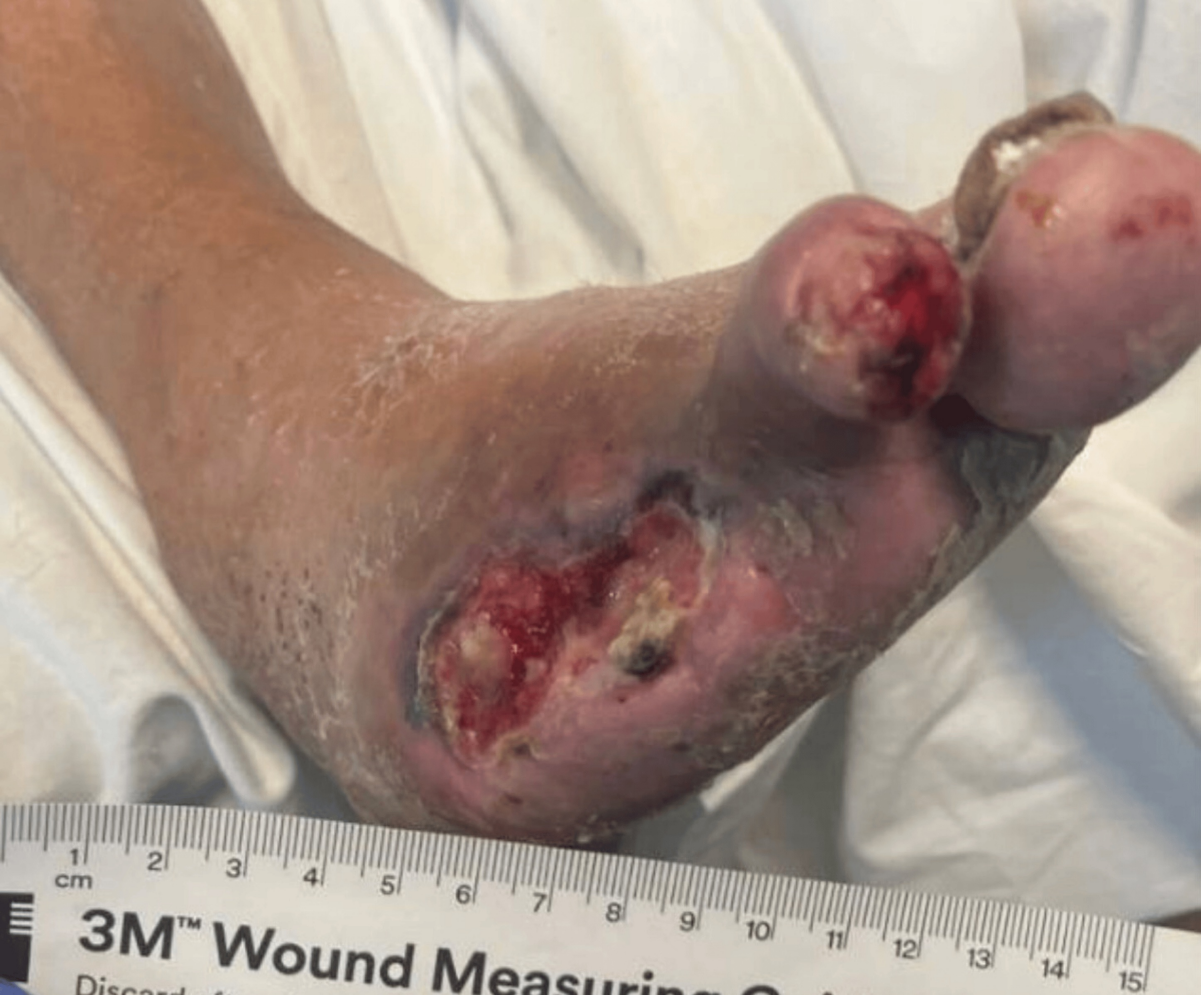
First Image of the Right Foot Right foot (lesion of approximately 5 cm length)

**Figure 2:**
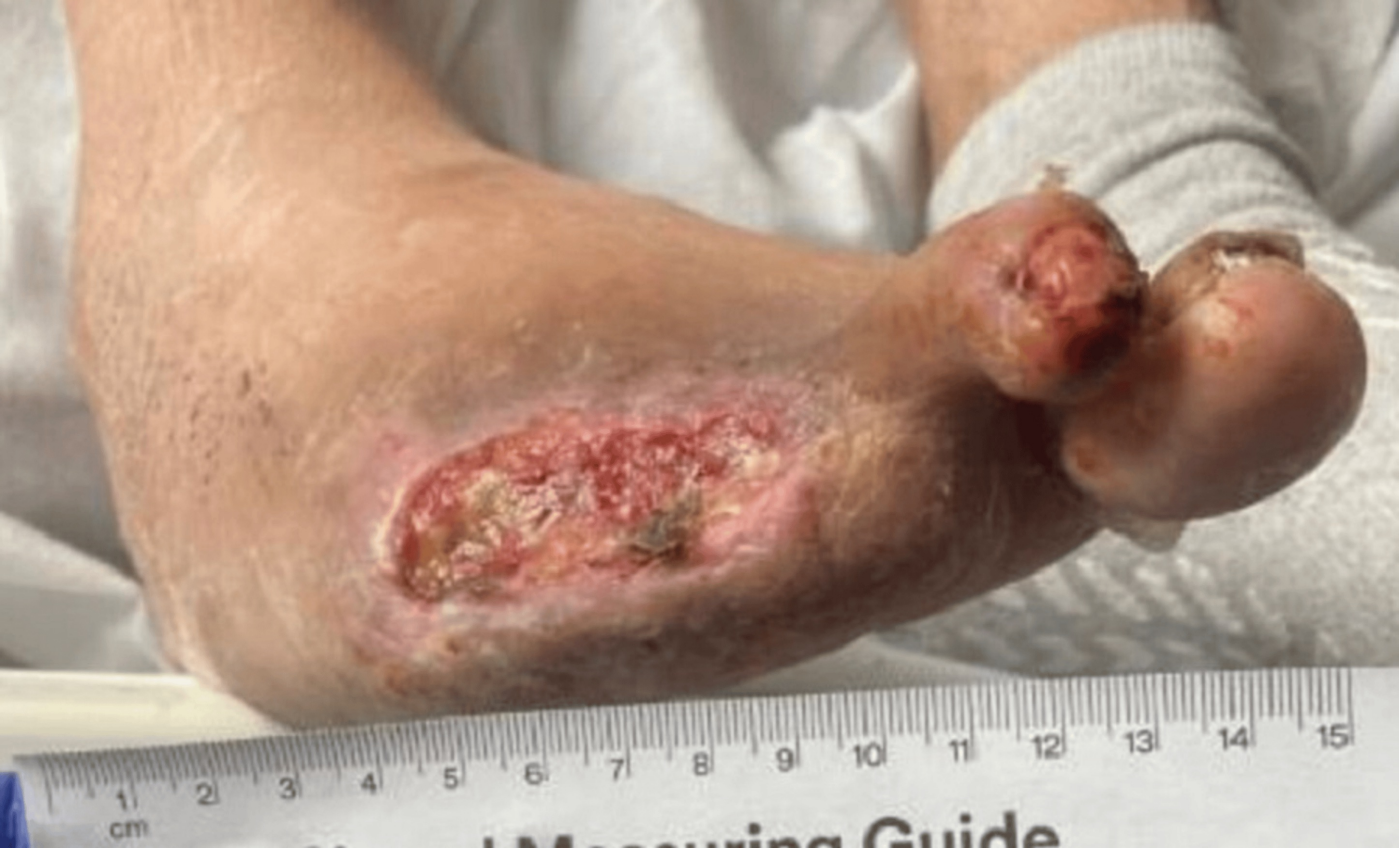
Second Image of the Right Foot Right foot (lesion of approximately 6 cm width)

**Figure 3:**
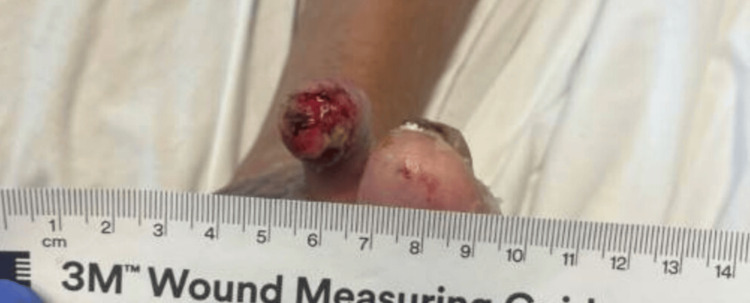
Image of the Right Second Toe

He had postoperative wounds that were complicated by peripheral artery disease and the non-healing of amputation sites. The patient had an ankle-brachial index (ABI) done, which showed a right toe index of 0.28 and a left toe index of 0.41, indicating severe peripheral artery disease. He was given a Rutherford classification of category 6 and grade III for major tissue loss extending above the transmetatarsal level with frank gangrene. Figures 1-3 show the images of the right foot used for classification.

**Figure 4:**
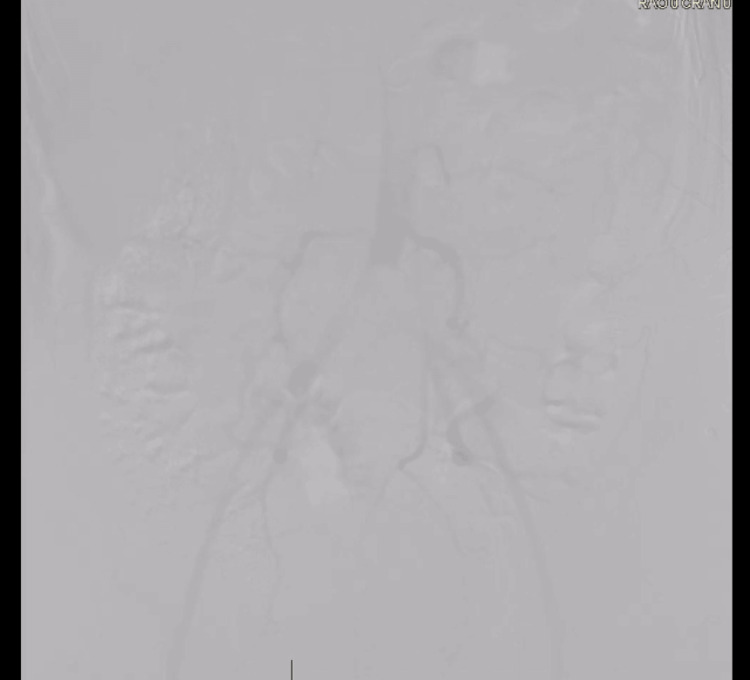
Occlusion of the Left CIA Occlusion of the left CIA, severe 99% stenosis of the right CIA, and distal occlusion just above the bifurcation of the abdominal aorta on peripheral angiography CIA: common iliac artery

**Figure 5:**
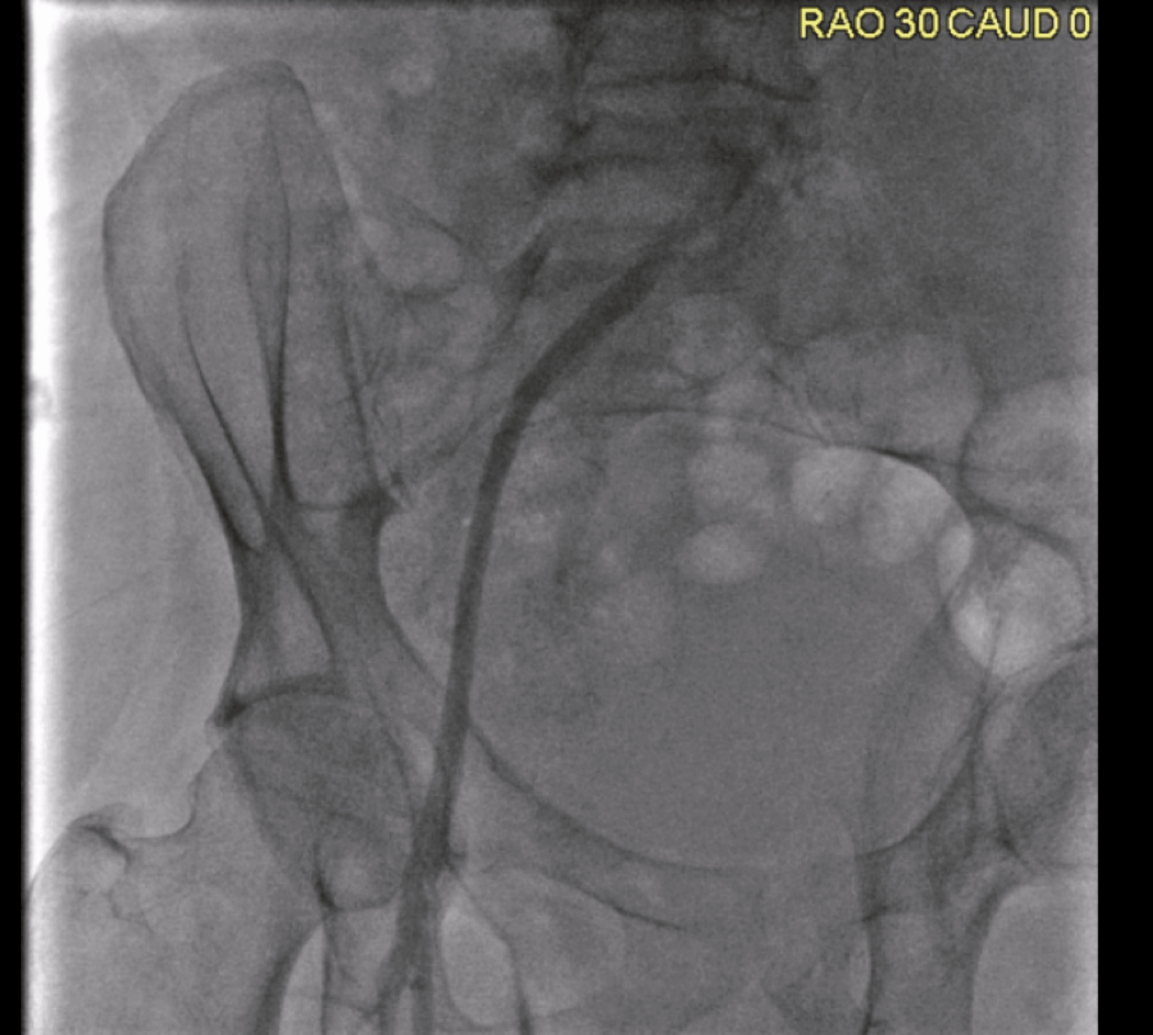
Right CIA Peripheral Angiography CIA: common iliac artery

**Figure 6:**
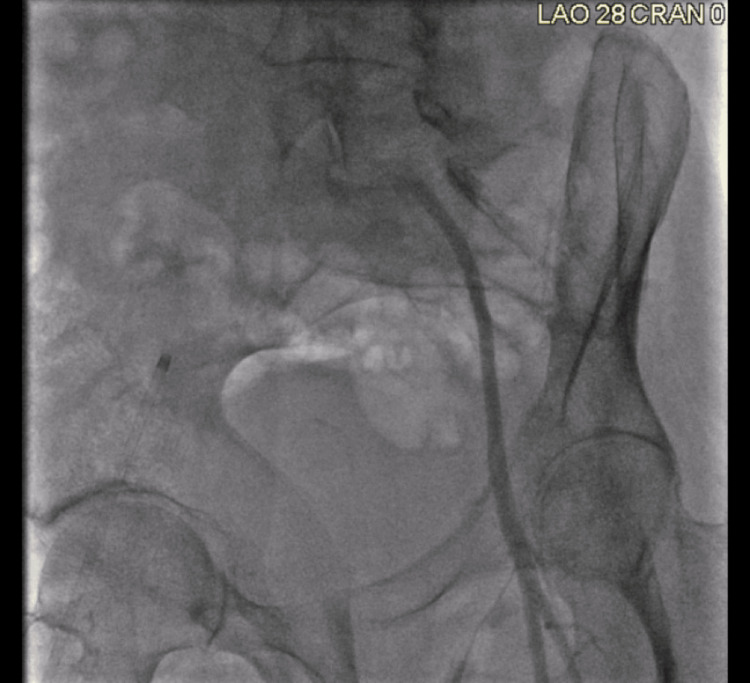
Left CIA Peripheral Angiography CIA: common iliac artery

The procedure to be done was a thrombectomy of bilateral CIA with Rotarex. Peripheral angiography showed occlusion of the left CIA, severe 99% stenosis of the right CIA, and distal occlusion just above the bifurcation of the abdominal aorta. Figures 4-6 and Videos 1-3 show the vascularity of the patient on angiography prior to intervention. Vascular access was obtained through left and right femoral access sites. Thrombectomy of the left CIA was attempted through the left femoral access site, and thrombectomy of the right CIA was attempted through the right femoral access site.

**Video 1:** Short Angiogram Cine Clip of Figure 4

**Video 2:** Short Angiogram Cine Clip of Figure 5

**Video 3:** Short Angiogram Cine Clip of Figure 6

**Figure 7:**
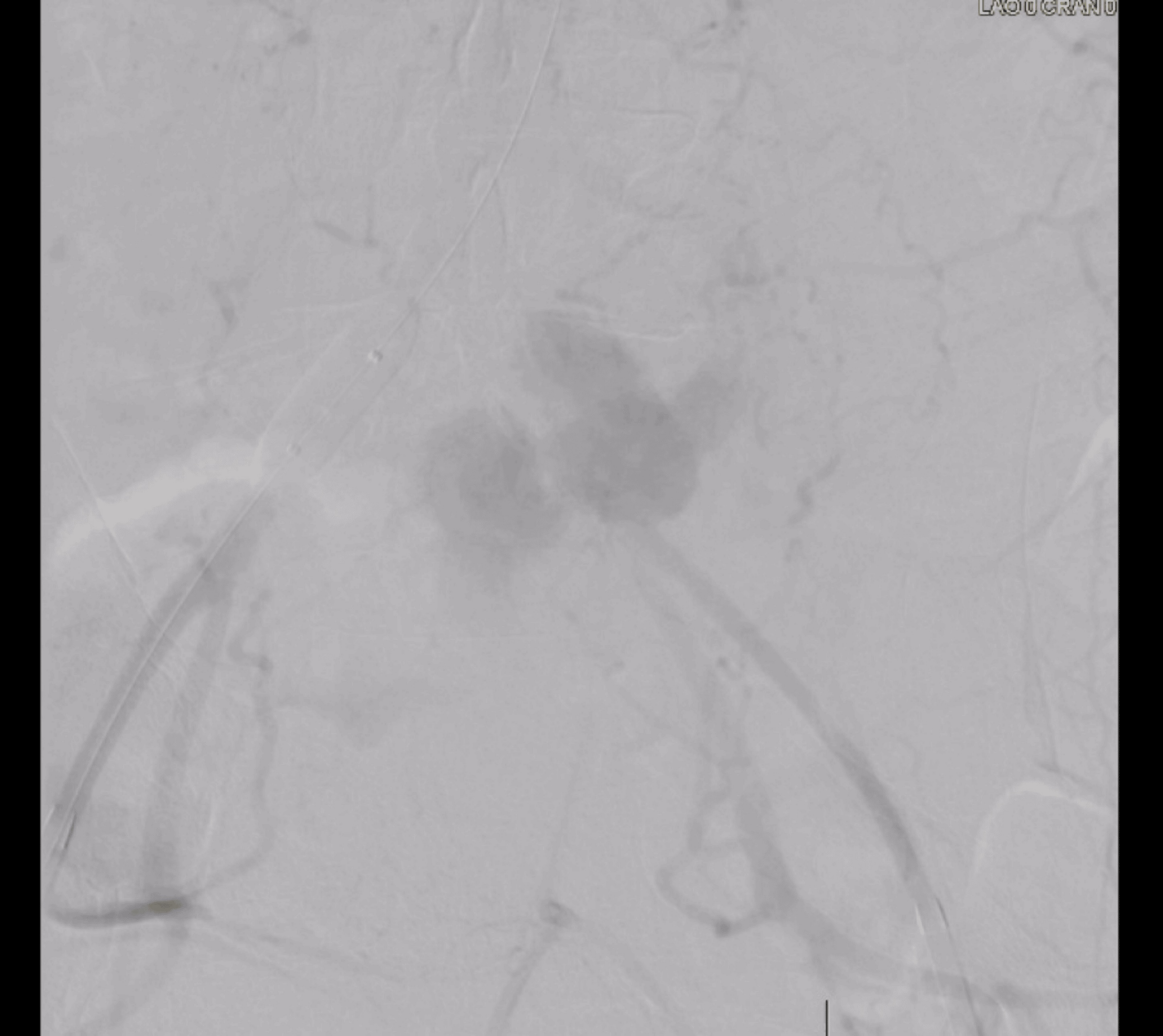
Extravasation of Blood Around the Left CIA CIA: common iliac artery

**Figure 8:**
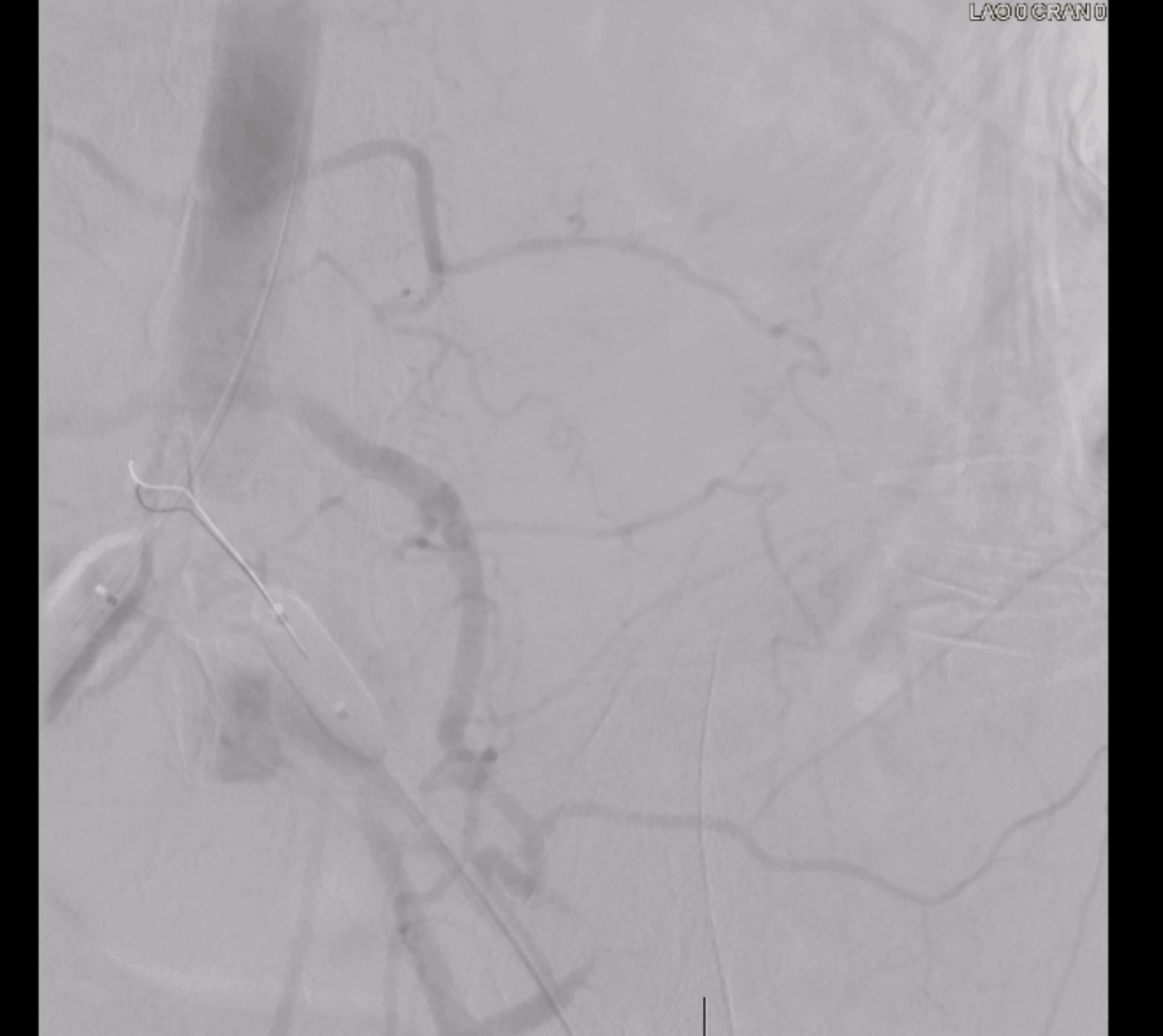
Extravasation of Blood Around the Left ICA, Abdominal Aorta Visualized CIA: common iliac artery

The device used was the 8F Rotarex mechanical thrombectomy system with an Armada balloon. Thrombectomy with stent placement was successful in the right CIA. On the left side, the device seized, which led to perforation of the left CIA, and the wire position was lost. It was not possible to rewire into the true lumen despite many attempts with multiple wires. Figures 7, 8 and Videos 4-7 demonstrate blood extravasation from the left CIA due to perforation seen on angiography. Prolonged balloon inflations were performed for several minutes until the surgical team could control the hemorrhage surgically. A 12 mm Armada balloon was inflated in the left CIA, and a 16 mm Armada balloon was inflated in the distal abdominal aorta. The patient was then transferred to the operating room for suture ligation. The surgical team asked for the balloons and wires to be removed before ligation, and a vascular clamp was placed distally in the left CIA to control blood flow. Placement was distal and not proximal, as this avoided further manipulation of the ruptured vasculature. Proximal placement would have required additional dissection, increasing the risk of blood loss and worsening hemodynamic status. During ligation, heavy calcifications were noted around the suture site. The operation was successful, and hemostasis was achieved. The patient was transferred to the Intensive Care Unit (ICU) for close monitoring.

**Video 4:** Short Angiogram Cine Clip of the Device Seizure and Perforation With Extravasation

**Video 5:** Short Angiogram Cine Clip of Figure 7

**Video 6:** Short Angiogram Cine Clip of Figure 8

**Video 7:** Short Angiogram Cine Clip of the Inflated Balloon With Surrounding Extravasation

In the ICU, an arterial duplex Doppler was performed for the left lower extremity. The results were reported in centimeters per second for each artery. They were as follows: Common Femoral Artery (CFA) proximal 32, Profunda Femoris Artery (PFA) proximal 32, Superficial Femoral Artery (SFA) proximal 35, SFA middle 35, SFA distal 19, Popliteal Artery proximal 14, Popliteal Artery distal 17, Anterior Tibial Artery distal 0, Dorsalis Pedis Artery 9, and Posterior Tibial Artery distal 9. The final impression stated the flow was slow throughout the left leg due to severe inflow disease with confirmed patencies of arteries to the popliteal artery. There was minimal flow below the knee with progressively slower flow in the distal tibial arteries. The CFA, PFA, and SFA had a parvus-tardus low flow, indicating upstream severe disease. There was only mild-moderate calcific disease identified in the CFA and SFA on 2D.

**Figure 9:**
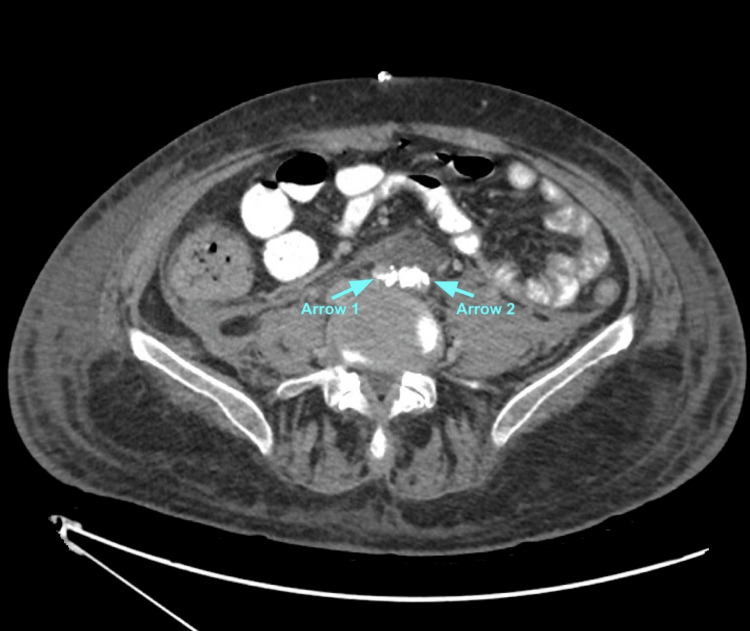
Pooling of Blood Due to Stenosis and Occlusion on CT Urogram Arrow 1: right CIA; Arrow 2: left CIA

**Figure 10:**
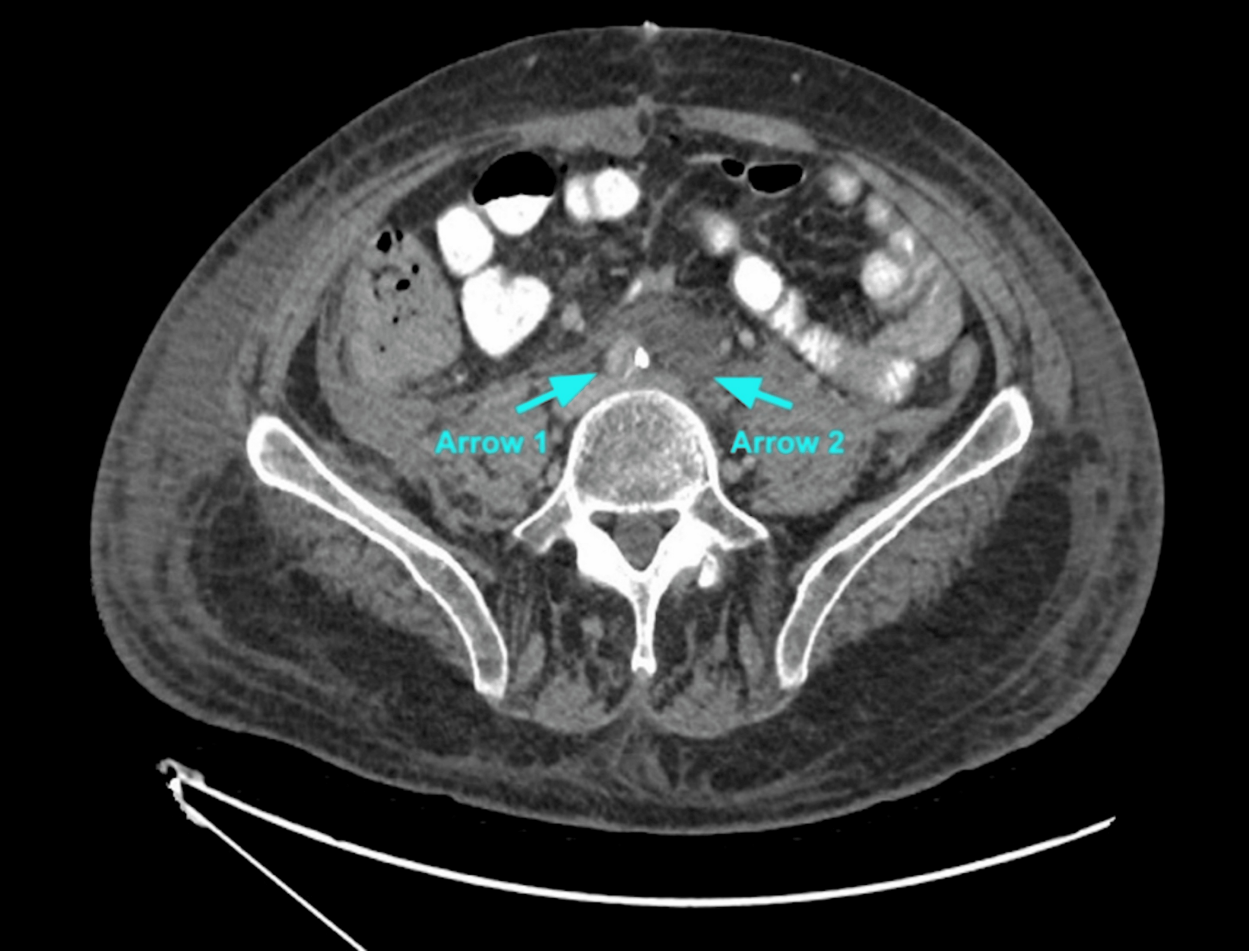
Severe Stenosis of the Right Common Iliac Artery and Occlusion of the Left Common Iliac Artery on CT Urogram Arrow 1: right CIA; Arrow 2: left CIA

The patient was noted to have red drainage in his urinary catheter, concerning for hemorrhage. Computed tomography (CT) urogram was done to rule out injury, and the only notable finding was the persistence of arterial occlusion. In Figure 9, an axial view of the CT shows blood pooling proximal to severe stenosis of the right CIA and occlusion of the left CIA. Figure 10 confirms these findings; the left CIA is attributable to prior occlusion seen on angiogram and ligation. Ultimately, the patient improved clinically, and he was extubated after an extended period of time. Considering his poor prognosis, goals of care discussions were initiated with the patient. After a thorough discussion, he opted for hospice with the goal of improving quality of life and avoiding further procedures.

## Discussion

For PTA, it is preferable to obtain access on the side where the thrombectomy is to be done, as it offers a more direct route to the target vasculature. In our case, the thrombectomy was bilateral, and respective femoral sites had been obtained for each side. Due to difficulty placing the wire into the true lumen, other percutaneous interventions, such as covered stents or embolization, were not used. This would require the precise location of the perforation to prevent further hemorrhage. Additionally, the patient had a large hematoma, was in critical condition on two inopressors, and required rapid transfusions, which prompted urgent intervention. Vasculature repair would require a longer operating time and is not as effective at stopping blood flow as ligation. Additionally, the patient had heavy calcification, which would have further complicated the likelihood of success with repair. Surgical bypass would have extended the time in the operating room and placed the patient at a greater risk of complications. He was hemodynamically unstable, requiring pressors at the time of intervention, which prevented the team from doing a surgical bypass.

In the left CIA, occlusion was already seen on the angiogram prior to ligation. Ligation of the left CIA and occlusion prevented normal blood flow to the left lower extremity. However, perfusion was maintained for this patient through cross-pelvic and collateral circulation. Through these mechanisms, blood flow from the right CIA and other arteries can indirectly reach the left lower extremity. In the physical exam, the bilateral dorsalis pedis and posterior tibial pulses were detected with Doppler ultrasound and were biphasic. Despite rupture and ligation, the presence of the distal pulses confirmed that perfusion was maintained despite aortoiliac occlusive disease. Alternative pathways, such as cross-pelvic and collateral circulation, allowed for blood flow to reach the distal left lower extremity. On arterial duplex Doppler, patent arteries with parvus-tardus waveforms indicated severe upstream disease. The flow, despite a proximal occlusion in the left CIA, demonstrated collateral circulation to the left lower extremity. The uniformity and manifestation of slow blood flow are evidence of alternative pathways supplying blood distally.

PTA was not successful, and the patient continued to have CLTI after the complication. Despite this, the patient was stabilized with emergent ligation in the operating room. Availability of the surgical team allowed for a prompt response to the complication. Data show that about half of peripheral interventions are performed by surgeons and the other half by radiologists and cardiologists [4]. This case serves as an example of the usefulness of surgical availability when PTA is done by nonsurgical professionals. It is also beneficial to examine the factors that put this patient at risk of complications. Particularly, his history of smoking and metastatic squamous cell cancer confer increased risk for morbidity and mortality. Former smokers have an increased postoperative risk of morbidity and mortality by approximately 20% [5]. Furthermore, postoperative mortality is significantly associated with metastatic cancer [6]. In patients with poor prognosis, consistent goals of care discussions improve outcomes. Specifically, patients with advanced non-small lung cancer who are enrolled in hospice require less acute care [7]. This supports the hospice discussions, which were initiated once the patient was stable enough for decision-making capacity.

As stated in the procedural documentation, a device malfunction led to the left CIA rupture. The device was seized on the wire in an area of heavy calcification, indicating entrapment. In PTA for complex lesions, the incidence of entrapment has been documented as approximately 1.5% [8]. The complexity of the lesion and the device are the direct contributors to the occurrence of aortoiliac rupture. With severe lesions, it is more difficult to advance and retrieve the device, which increases the risk of rupture. The literature establishes that smaller vessels, such as the EIA, are more likely to rupture during PTA [3]. However, this case involves rupture of a larger vessel - the left CIA. With arterial plaque involvement, there are other factors to consider in addition to the size of the vessel. These include location within the vascular wall as well as volume and shape of the plaque. For example, larger volume, irregular shapes, and superficial plaques all increase risk for rupture [9]. Visualization with the naked eye during the ligation procedure showed a superficial plaque with large volume. This is thought to have directly influenced the occurrence of rupture despite involvement of a larger vessel in this case. A consideration that may have allowed for better appreciation of the lesion is referred to as preprocedural cross-sectional imaging (PCSI). This gives the proceduralist an opportunity to preemptively plan better for revascularization. PCSI has associated risks such as radiation and increased contrast load, which may limit its usefulness depending on the context. A study from Vascular and Endovascular Surgery examined PCSI, concluding its use generally had no influence on outcomes for PTA [10]. The complication in this case introduces the potential benefit of PCSI in special circumstances. The use of this imaging offers insight into lesion extensity, assists with procedural planning, and is hypothesized to result in better patient outcomes.

Procedure-wise, PTA with selective stenting is one of the various techniques that may be considered. Broadly, other procedures include primary stenting, surgical grafting, atherectomy, cutting balloons, and lithotripsy. Beginning with primary stenting, the procedure is not as cost-effective as PTA with selective stenting [1]. Stent placement is often not needed; however, primary stenting may be preferred in more severe cases [11]. Furthermore, in the highest degree of severity, surgical grafting is considered in accordance with the surgical risk of the patient, as it is generally more effective [12]. Atherectomy directly removes plaques and has shown some benefit, yet there is limited data for the treatment of aortoiliac disease [13]. Cutting balloons penetrate plaques before balloon inflation; they have proven useful in certain cases of coronary stenting [14]. However, limited data exist to support its usefulness in the context of aortoiliac disease. The last procedure, lithotripsy with the Shockwave L6 balloon, is a novel technique and uses shock waves to break up calcified lesions. Initial reports have shown that this new technology is acceptable for aortoiliac disease and can prepare vessels prior to PTA [15,16]. All of the procedures discussed may be considered for the treatment of iliac disease. PTA with selective stenting may be chosen as a more cost-effective treatment of less severe lesions. Conversely, surgical grafting and primary stenting may be used as alternatives for patients with severe disease. Another acceptable alternative is lithotripsy; though, due to its novelty, there is not enough clinical data for any conclusive suggestions. Data show that lithotripsy may be safe for preparing vessels for PTA and can be used in conjunction with PTA.

The key points from this case involve understanding the extent of the plaque and the specific device that led to the complication. The large volume and superficial nature of the plaque created an environment that impeded the ability to navigate the device. After rupture, surgical capabilities at the facility achieved resolution of the complication. As a substantial amount of PTA procedures are done by nonsurgical professionals, it is ideal to have a multidisciplinary team available in case of complications. In hindsight, implementation of PCSI may have provided insight into the extent of the lesion and guided treatment. With severe stenosis, there are additional procedures to consider for revascularization, such as primary stenting, surgical grafting, and lithotripsy. The importance of this case is underscored by awareness, understanding of the precipitating factors, and knowledge of preventive strategies.

## Conclusions

This report analyzes a case of CIA rupture during PTA with selective stenting. While this procedure is widely implemented and generally benign, the availability of a surgical team is ideal in cases of complications. The extent of plaque and the device implicated provide insight into the challenges of endovascular intervention. This case exemplifies the characteristics of large volume and superficiality, which increase the risk of plaque rupture. For severe lesions, PCSI and alternatives to PTA, namely primary stenting, grafting, and lithotripsy, may be considered for revascularization. Evaluation of complex lesions in at-risk patients and attention to alternative therapies are key to preventing recurrence of this complication.
